# A modular microfluidic platform to study how fluid shear stress alters estrogen receptor phenotype in ER^+^ breast cancer cells

**DOI:** 10.21203/rs.3.rs-3399118/v1

**Published:** 2023-10-10

**Authors:** Braulio Andrés Ortega Quesada, Jonathan Cuccia, Rachael Coates, Blake Nassar, Ethan Littlefield, Elizabeth C. Martin, Adam T. Melvin

**Affiliations:** 1 Cain Department of Chemical Engineering, Louisiana State University, Baton Rouge, LA, 70803; 2 Department of Chemical and Biological Engineering, Clemson University, Clemson, SC, 29634; 3 Biological and Agricultural Engineering, Louisiana State University, Baton Rouge, LA, 70803; 4 Department Medicine, Section Hematology and Medical Oncology, Tulane University, New Orleans, LA, 70118

**Keywords:** metastasis, single cell resolution, ER^+^ breast cancer, endocrine therapy, microfluidics, fluid shear stress

## Abstract

Metastatic breast cancer leads to poor prognoses and worse outcomes in patients due to its invasive behavior and poor response to therapy. It is still unclear what biophysical and biochemical factors drive this more aggressive phenotype in metastatic cancer; however recent studies have suggested that exposure to fluid shear stress in the vasculature could cause this. In this study a modular microfluidic platform capable of mimicking the magnitude of fluid shear stress (FSS) found in human vasculature was designed and fabricated. This device provides a platform to evaluate the effects of FSS on MCF-7 cell line, a receptor positive (ER^+^) breast cancer cell line, during circulation in the vessels. Elucidation of the effects of FSS on MCF-7 cells was carried out utilizing two approaches: single cell analysis and bulk analysis. For single cell analysis, cells were trapped in a microarray after exiting the serpentine channel and followed by immunostaining on the device (on-chip). Bulk analysis was performed after cells were collected in a microtube at the outlet of the microfluidic serpentine channel for western blotting (off-chip). It was found that cells exposed to an FSS magnitude of 10 dyn/cm^2^ with a residence time of 60 seconds enhanced expression of the proliferation marker Ki67 in the MCF-7 cell line at a single cell level. To understand possible mechanisms for enhanced Ki67 expression, on-chip and off-chip analyses were performed for pro-growth and survival pathways ERK, AKT, and JAK/STAT. Results demonstrated that after shearing the cells phosphorylation of p-AKT, p-mTOR, and p-STAT3 were observed. However, there was no change in p-ERK1/2. AKT is a mediator of ER rapid signaling, analysis of phosphorylated ERα was carried out and no significant differences between sheared and non-sheared populations were observed. Taken together these results demonstrate that FSS can increase phosphorylation of proteins associated with a more aggressive phenotype in circulating cancer cells. These findings provide additional information that may help inform why cancer cells located at metastatic sites are usually more aggressive than primary breast cancer cells.

## Introduction

Breast cancer is one of the most commonly occurring cancers with one in eight women in the United States diagnosed annually and greater than 285,000 new cases reported in 2022 alone.[1] Of these newly diagnosed breast cancer cases, approximately 70% are hormone receptor, estrogen receptor-positive (ER^+^) and progesterone receptor-positive (PR^+^).[2, 3] Many ER^+^ breast cancer patients are given a poorer prognosis following metastasis to secondary sites. Approximately 90% of all cancer-related deaths are associated with metastatic spread since secondary tumors are usually more resistant to therapy and have a higher proliferation rate.[4, 5] Metastasis occurs through a cascade of five distinct steps: invasion, intravasation, circulation, extravasation, and colonization.[6] During the circulation step through the vasculature, metastasizing breast cancer cells are exposed to a wide range of chemical and physical cues that can result in both genotypic and phenotypic changes leading to altered behavior and increased aggressiveness at the metastatic site. There are several mechanisms to explain why metastatic tumors become more aggressive including one that postulates that the transition from classical estrogenic signaling of ER^+^ breast cancer to a growth factor signaling pathway facilitates the observed aggressiveness at metastatic sites. More recently, studies have identified alternative activation of classically growth factor mediated signaling cascades through mechanical stimuli. One of the major mechanical cues affecting the cells during metastasis is exposure to fluid shear stress (FSS).[7] FSS can be defined as the internal frictional force between moving layers in laminar flow and can be understood as the force perpendicularly applied per unit area on a surface.[4] While several studies have suggested a role for the forces a cell experiences while in vascular flow, FSS, in creating more aggressive metastatic tumors, the underlying genotypic and phenotypic changes that are altered when ER^+^ breast cancer cells are exposed to FSS have not been fully elucidated. Cells within the body are exposed to interstitial shear stress which can be present in tumor tissue at rates around 0.1 dyn/cm^2^. Cells flowing through the vasculature including veins, capillaries, and arteries are exposed to FSS with magnitudes of 1–4 dyn/cm^2^, 10–20 dyn/cm^2^, and 4–30 dyn/cm^2^, respectively.[4] Lee et al. demonstrated that interstitial shear stress could promote tumor invasion and metastasis, adhesion, and extravasation after exposure at rates of 0.05 dyn/cm^2^ in human prostate cancer cell lines.[8] Some studies have reported that after exposure to FSS, MCF-7 cells presented a significant increase in the cancer stem cell population.[9] Chang et al. found that after exposing cancer cells to FSS at 12 dyn/cm2 there is G2/M arrest inhibiting cell differentiation.[10] Furthermore, pro-growth and survival pathways typically mediated by growth factors, such as ERK and AKT, are observed to be enhanced following FSS.[11–14] However, it is currently unknown if the observed protein phosphorylation is due solely to exposure to FSS or in tandem with exposure to extracellular growth factors. Because of this, there is a need to understand how FSS may affect activation of pro-survival and proliferation pathways.

Several models and approaches have been designed to mimic and monitor the mechanical cues, including FSS, caused by circulation on metastasizing cancer cells. Triantafillu et al. used polyether ether-ketone (PEEK) tubing connected to a syringe pump to expose cells to FSS; [9] whereas Choi et al., cultured cells in an orbital shaker to mimic the shear stress exerted during circulation.[15] Some groups have used peristaltic pumps connected to tubing to expose the cells to the FSS in a closed system[16] while other studies have implemented a parallel-plate flow chamber in which they mimic the wall shear stress in laminar flow.[17, 18] Unfortunately, some of these larger volume systems allow for greater fluctuations in the magnitude of FSS exerted on individual cells which can introduce more variability across the population of sheared cells when studying cells at the single cell level. This is due to the variation in FSS magnitude depending on the position of the cell in the cross-sectional area of the system due to the velocity profile in a tube being parabolic. Therefore, the position of the cells in the volume is an important parameter that needs to be tightly controlled to ensure that the FSS experienced by each cell is consistent. The variability of FSS experienced by the cells using these methods (sometimes across several orders of magnitude) suggests that observed biological differences could be attributed to subpopulations of cells being exposed to varying magnitudes of FSS potentially biasing the findings. Moreover, these bulk analysis methods may mask small populations of cells resulting in distinct subpopulations of cells that are (or are not) affected by fluid shear stress being overlooked.[19] This motivates the need for a system capable of exposing cells to uniform FSS magnitudes that is also able to observe FSS-induced changes at the single cell level. Some microfluidic platforms have been fabricated to study how FSS affects cancer cells, for example Ni et al. developed a Plug and Play platform in which they tried to recreate each metastasis stage.[20] Dash et al. created a microfluidic device in which they exposed HeLa cells to a wide range of physiological FSS magnitudes.[21] A device generated by Landwehr et al. demonstrated that individual ER^+^ (MCF-7) and triple negative (MDA-MB-231) breast cancer cells respond differently when exposed to the same magnitudes and durations of FSS with some cells exhibiting greater deformability as the FSS rate and duration increased motivating the need for single cell studies.[22] Many *in vitro* models for single cell analysis have been developed and have been categorized by their cellular isolation or trapping methodologies. Single cell isolation devices have been classified into four approaches: microposts, microfiltration, microwells, and trapping chambers.[23] With its high throughput, ease of manufacturing, and utilization of gravity and cell size to capture cells, the microwell approach is the easiest to operate. This method also has the advantage that cell trapping is not highly dependent on a control system to regulate the pumps governing flow conditions in the device. Furthermore, in microwells, cells are confined to private and uniform environments enabling precise control of cell or cluster shape and size.[24]

A modular microfluidic platform consisting of both a shearing module and microwell trapping array was designed and fabricated to expose individual cells to an approximately uniform magnitude of FSS to investigate how exposure to shear results in phenotypic changes in ER^+^ breast cancer. This approach is essential to ensure any changes in cellular phenotype can be attributed exclusively to the response of each cell to the specific treatment and not due to fluctuations in the treatment itself. The shearing device was designed to be modular so that it could be utilized in multiple ways. Cancer cells exiting the shearing module could be either collected in microcentrifuge tubes and analyzed by standard laboratory techniques such as western blot (herein referred to as off-chip analysis) or the cells could be flowed into the microwell trapping array for single cell immunostaining (herein referred to as on-chip analysis). On-chip analysis results indicated that exposing MCF-7 cells to 10 dyn/cm^2^ FSS caused an increase in Ki67 expression, a biological marker associated with enhanced proliferation, in the sheared population over the non-sheared population. These findings suggest that FSS plays a role in inducing more proliferative cellular behavior. Both on-chip and off-chip analysis showed enhanced AKT phosphorylation after being exposed to FSS. Similarly, phosphorylation of Activation of Signal Transducer and Activator of Transcription-3 (STAT3) and mTOR was observed to be enhanced in cells exposed to shear compared to non-sheared controls; however, on-chip and off-chip analysis showed no change in the phosphorylation of ERα between the sheared and non-sheared controls. This study demonstrates that a physiological magnitude of FSS is capable of inducing phenotypic changes in growth factor signaling pathways and enhancing proliferative biomarkers in shear exposed ER^+^ breast cancer cells.

## Results and Discussion

### Computational characterization of velocity and shearing profiles in the modular microfluidic device

**Figure 1.**
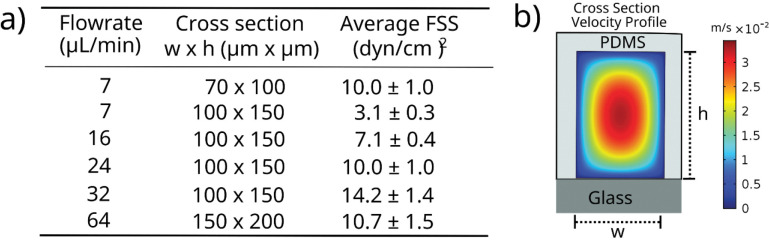
COMSOL simulation and fluid shear stress magnitudes in different shearing modules. **a**) Table showing the dependence of the fluid shear stress magnitude with different dimensions of the cross-sectional area of the channel of the shearing module at different flowrates. **b**) Velocity profile of the 100 × 70 μm cross-sectional area shearing module at 7 μL/min.

The purpose of the modular microfluidic platform was to expose single cells to approximately uniform magnitudes of shear. This approach allowed for precise control in forces exerted on individual cells so that any observed single cell heterogeneity could be attributed to inherent differences in the population of cells and not to differences in how the cells were interrogated. To achieve this, several iterations in the dimensions of the cross-sectional area of the shearing device were simulated using COMSOL Multiphysics to minimize the differences in FSS magnitude applied to the cells at different positions in the channel (e.g., in the center versus near the wall). Four different combinations of channel width (70–150 μm) and height (100–200 μm) were investigated at five different flow rates (7–64 μL/min) (Figure 1A). The rationale for studying the different combinations (and different cross-sectional areas) was to allow for studies at different FSS magnitudes while maintaining the same residence time of ~60 s in the device while minimizing the pressure drop across the device. As expected, FSS magnitude applied to the cells was dependent on the flowrate and the dimensions of the cross-sectional area of the channel. For example, a volumetric flowrate of 7 μL/min resulted in a 69% decrease in FSS magnitude when the cross-sectional area increased from 70 × 100 μm to 100 × 150 μm (Figure 1A). Cells can be exposed to varying FSS magnitudes when transported through the vasculature spanning 5–60 dyn/cm^2^; however, this work focused on studying an average FSS magnitude of 10 dyn/cm^2^, which is a physiologically representative rate found in capillaries and arteries.[4] The simulations indicated that cross-sectional areas 70 × 100 μm (with a flowrate of 7 μL/min) or 100 × 150 μm (with a flowrate of 24 μL/min) achieved the desired setpoint with an average fluid velocity of 1.8 ± 0.2 cm/s (Figure 1A). As expected, a velocity gradient was observed across the cross-sectional area with a maximum value in the center of the channel and a minimum near the wall (Figure 1B). An advantage of the microfluidic approach was the ability to minimize the fluctuations in FSS magnitudes applied to the cells as each geometry/flowrate combination resulted in only a 10% difference in velocity for the 70 × 100 μm channel and a 10% difference for the 100 × 150 μm channel. As such, each cell was expected to be subjected to similar FSS magnitudes, something that is difficult to control in other systems with larger cross-sectional areas. It was determined that this percent change in FSS magnitude did increase with increasing cross-sectional areas and volumetric flowrates achieving a maximum of 16% using the combination of 64 μL/min in the 150 × 200 μm channel (Figure 1A). This means that studies involving larger FSS magnitudes will result in greater heterogeneity of the forces exerted on the cells. Additionally, the system was also confirmed to work with laminar flow with a calculated Reynolds number on the order of 10^−5^ and the FSS magnitude being longitudinally constant. Similar COMSOL simulations were performed on the microwell trapping array to ensure that the trapped single cells were not exposed to additional FSS during washing and immunostaining steps thus biasing the findings between the on-chip and off-chip studies. These simulations demonstrate that the FSS profile on the trapped cells is sufficiently low (10^−5^ dyn/cm^2^), proving negligible FSS exposure outside of the shearing device (Figure S1).

It was also important to calculate the pressure drop across the device in addition to simulating the velocity profiles. This pressure drop was calculated using a variant of the Hagen-Poiseuille equation for rectangular channels (see supporting information) and it was found to be 70 kPa in the 70 × 100 μm channel with a total length of 1m. The pressure drop increased as a function of channel length rising to 105 kPa for a 1.5m long channel and 140 kPa for a 2m long channel. These larger pressure drops would make flowing fluid through the device increasingly challenging using a syringe pump without the inlet tubing becoming displaced due to back pressure. As such, it was determined to increase the cross-sectional area of the channel and the channel length to achieve higher magnitudes of FSS with similar residence times. Therefore, it was decided to use the 70 × 100 μm device to shear at rates of 10 dyn/cm^2^ and the 100 × 150 μm device to shear at 20 dyn/cm^2^ since the latter needed to be longer to maintain the same exposure time (regarding that the velocity would increase to reach the 20 dyn/cm^2^).

### Exposure to FSS induces the expression of a proliferative marker in single MCF-7 cells.

**Figure 2.**
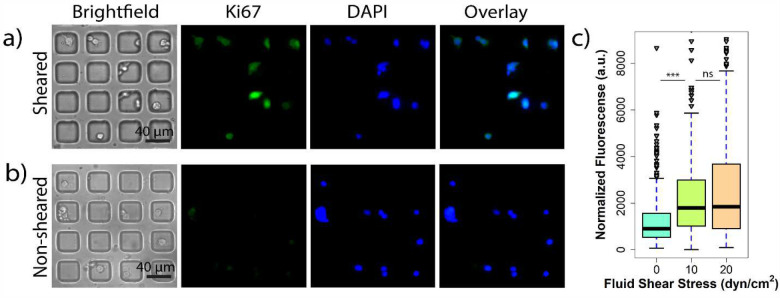
On-chip analysis of MCF-7 cells exposed to FSS exhibit enhanced Ki67 protein levels as a marker of proliferation. **a**) Representative immunostaining images of single MCF-7 cells exposed to shear at a magnitude of 10 dyn/cm^2^ for Ki67 (proliferation, green) and DAPI (nuclear, blue). **b**) Representative immunostaining images of single non-sheared MCF-7 cells for Ki67 (proliferation, green) and DAPI (nuclear, blue). **c**) Quantification and distribution of normalized fluorescence in single cells coupled with one-way ANOVA which shows statistically significant changes between the sheared population and non-sheared population (*** indicates statistically significant p<0.001, ns indicates statistically non-significant p>0.05).

Prior studies have shown that exposure to FSS alters the phenotype of ER^+^ breast cancer cells which could lead to enhanced rates of proliferation.[9, 25] The first set of experiments using the modular microfluidic system were to further investigate this hypothesis to determine if exposure to FSS results in enhanced expression of markers of proliferation. Ki67 is a standard marker used in oncology as a measure for proliferation in cancer cells[26] with high levels of Ki67 in breast tumors linked to enhanced proliferation, aggressiveness, and a poorer prognosis.[27, 28] On-chip experiments were performed exposing MCF-7 cells to two different FSS magnitudes (10 and 20 dyn/cm^2^) in addition to a no shear control. A clear difference was observed in Ki67 expression between cells exposed to 10 dyn/cm^2^ FSS and 0 dyn/cm^2^ (Figure 2A–B). Interestingly, while there is a statistical difference between the non-sheared population and both sheared populations, there is no statistical difference between the populations of cells exposed to either 10 or 20 dyn/cm^2^ FSS (Figure 2C). This indicates that exposing cells to a higher FSS magnitude does not significantly alter the expression of the proliferative marker Ki67. The distribution of the single cell Ki67 expression found that exposing cells to higher magnitudes of FSS resulted in substantially higher degrees of heterogeneity. Cells exposed to 10 dyn/cm^2^, and especially 20 dyn/cm^2^, resulted in small subpopulations of extremely high Ki67 expression in the fourth quartile with some outliers with even higher expression (Figure 2C). This finding is in line with prior studies by Landwehr et al suggesting that single cells respond differently to exposure to FSS which could potentially explain why some CTCs survive in the vasculature while others do not.[22] Overall, the results from the Ki67 studies suggest that exposure to FSS enhanced the expression of the proliferation marker Ki67 immediately after FSS exposure.[4]

### Cells exposed to FSS activate cell proliferation and survival through AKT and mTOR phosphorylation.

Prior work has shown that phosphorylation of ERK and AKT are linked to enhanced tumorigenesis, cell survival, and cell proliferation.[29–33] Based on the findings in Figure 2, it was suspected that the enhanced markers of proliferation in sheared ER^+^ breast cancer cells was due to enhanced activation of pro-proliferative pathways such as ERK. To investigate this, MCF-7 cells were exposed to 10 dyn/cm^2^ magnitude of FSS in addition to a no shear control and then interrogated for alteration in p-ERK1/2 (Thr202/tyr204) levels using both on-chip and off-chip experimentation. Opposed to what was expected both sheared and non-sheared populations of cells did not exhibit a statistically significant difference in ERK1/2 (Thr202/Tyr204) phosphorylation (Figure S2). The median fluorescence intensity of both populations was found to be nearly identical (around 1200, Figure S2B) with the first and second quartiles being equal, and the fourth quartile of the sheared population being barely larger than the same quartile of the non-sheared population. These findings suggest that exposure FSS does not affect the phosphorylation of ERK1/2 (Thr202/Tyr204) under the conditions of this study (1 min of exposure at 10 dyn/cm^2^). This may be due to multiple other factors such as 10 dyn/cm^2^ FSS may preferentially activate other pathways, or 10 dyn/cm^2^ may be too low of a magnitude to be an activating stimulus for ERK1/2 in MCF-7 cells. It is also possible that a longer residence time in the device (> 1 min) is required to induce ERK1/2 phosphorylation in this cell line. Other studies have found that exposure times higher than 10 minutes under similar FSS magnitudes are capable of activating p-ERK1/2 in HUVEC and liver cancer stem cells, indicating that ERK1/2 may require more exposure time to phosphorylate.[11, 12]

**Figure 3.**
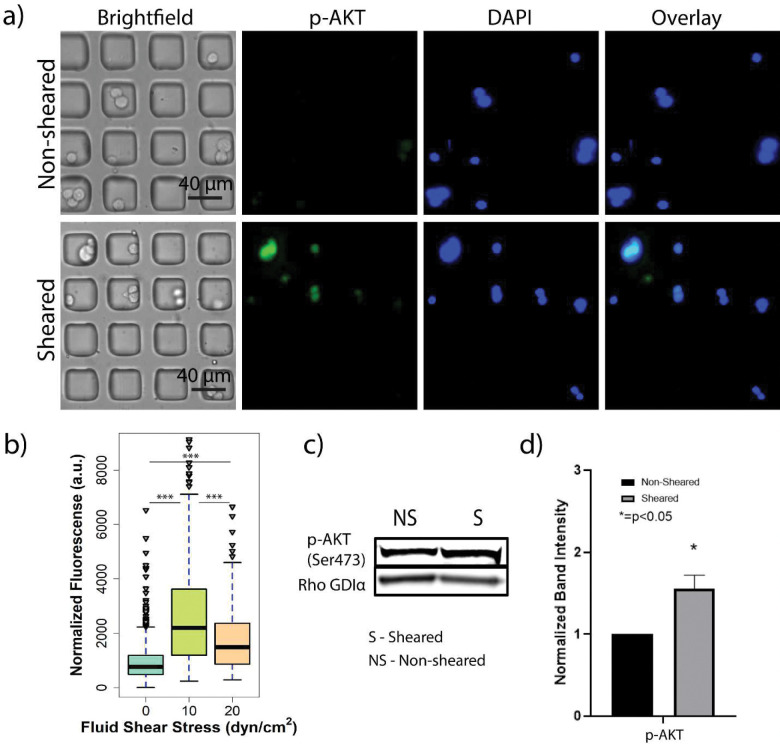
Exposure to FSS results in AKT phosphorylation in MCF-7 cells in both on-chip and off-chip experiments. **a**) Immunostaining images of AKT phosphorylation (p-AKT, green) and DAPI (nuclear, blue) in non-sheared and sheared cells at 10 dyn/cm^2^ **b**) Quantification and distribution of normalized fluorescence in single cells coupled with one-way ANOVA which shows statistically significant changes between the sheared population and non-sheared population. **c**) Western blot assay for p-AKT (Ser473) **d**) Graphical representation of the normalized band density. The amount of phosphoprotein was normalized to Rho GDIα, and amount of phosphoprotein from the non-Sheared population was standardized at 1. Data is representative of n=2 biological replicates, mean ± SEM. (*** indicates statistically significant p<0.001, * indicates statistically significant p<0.05, ns indicates statistically non-significant p>0.05).

Under the hypothesis that other pathways for proliferation and survival may be activated after exposure to FSS, it was of interest to study the expression of AKT following FSS. A clear difference in the cellular fluorescence was observed in single MCF-7 cells between the sheared (10 dyn/cm^2^) and non-sheared populations suggesting that exposure to FSS is able to enhance AKT phosphorylation (Figure 3A). Figure 3B shows that non-sheared cells (0 dyn/cm^2^) exhibit a statistically different distribution of p-AKT when compared with the populations exposed to FSS magnitudes of 10 and 20 dyn/cm^2^. Moreover, the analysis yielded a different finding when compared to the Ki67 staining with MCF-7 cells exposed to 20 dyn/cm^2^ FSS exhibiting diminished AKT phosphorylation with 20 dyn/cm2 when compared to cells exposed to 10 dyn/cm^2^ FSS (Figure 3B). The cell population exposed to 10 dyn/cm^2^ shows a third quartile that is higher than the third quartile of the population exposed to 20 dyn/cm^2^ and the outliers of this population would fit in the fourth quartile of the population sheared to 10 dyn/cm^2^. This finding suggests that 10 dyn/cm^2^ is sufficient to activate p-AKT. These findings also suggest possible mechanisms for the observed presence of p-AKT in metastatic sites. This is supported by recent work by Tokunaga et al found that, from 36 metastatic breast cancer cases of which 33 were ER^+^, 12 of those were p-AKT-positive and showed worse efficacy when treated with endocrine therapy when they were compared to p-AKT-negative patients.[34]

Off-chip western blot analysis of AKT phosphorylation was then performed to complement the on-chip immunostaining experiment. The off-chip analysis observed similar findings to the on-chip studies with a significant difference (p<0.05) of a 1.5 ± 0.2-fold increase in p-AKT levels in the sheared (10 dyn/cm^2^) population when compared to the non-sheared control (Figure 3C–D). Interestingly, the differences in p-AKT levels between the sheared and non-sheared populations was found to be greater in the on-chip experiments when compared to the off-chip experiment. To investigate this, the number of cells that were positive for the immunostaining of p-AKT (on-chip) were counted and compared to the number of cells that were counterstained for DAPI to determine if that could explain the difference between the on-chip and off-chip results. It was found that the total amount of cells in the sheared population had a larger proportion of p-AKT-positive cells (43%) when compared to the non-sheared population of p-AKT positive cells (26%) (Figure S3).

This may be since, without stimulation, p-AKT has a base-line level in bulk analysis and until an external stimulus, or a growth factor binds to the membrane receptor of the cell, that level increases. In such a case, FSS is acting as an external stimulus which enhances the phosphorylation of AKT, and so, a larger proportion of AKT phosphorylated cells are detected with a higher p-AKT expression in the sheared population. An analysis on the distribution of the fluorescence signal for both populations found that members of the non-sheared population expressed generally lower intensities (corresponding to AKT phosphorylation) when compared to the sheared population with the majority of cells having fluorescence signals barely above the baseline (66% of the total non-sheared population have normalized fluorescence intensities under 1000, Figure S4). This single cell analysis suggests that the driving force for the observed difference in changes in AKT phosphorylation between the on-chip and off-chip experiments results from two distinct subpopulations of cells: one population of non-sheared cells that fail to show any AKT phosphorylation and one population of sheared cells with very high level of AKT phosphorylation. This finding supports the importance of performing single cell analysis since the bulk analysis of lysates can mask distinct subpopulations that exhibit a different phenotype which could correspond to enhanced pathway activation due to exposure to FSS. These subpopulations of cells with enhanced AKT phosphorylation could overlay with the population of cells that exhibit enhanced markers of proliferation and could represent the number of metastasizing cells that survive exposure to FSS in the vasculature.

**Figure 4.**
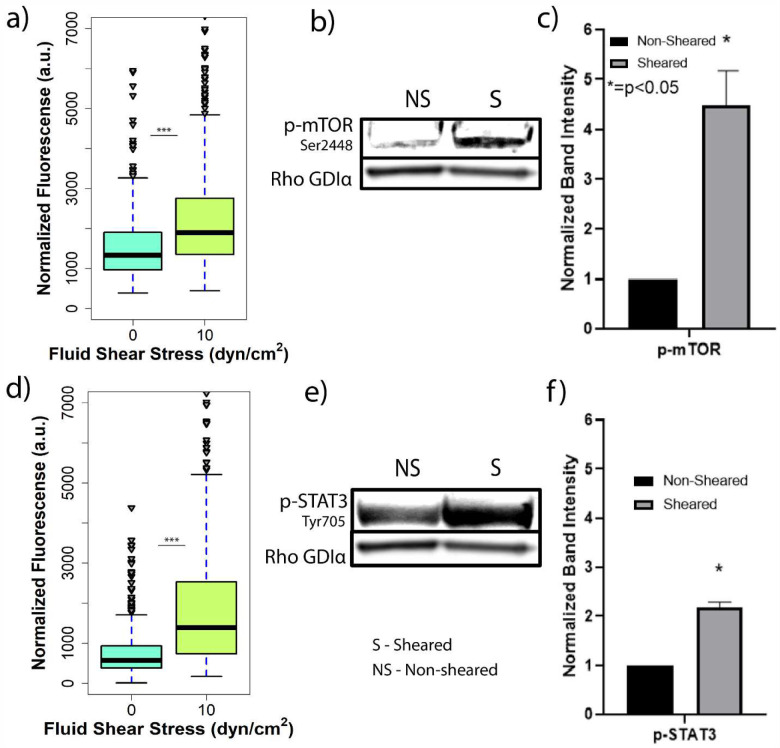
Enhanced phosphorylation of mTOR and Signal Transducer and Activator of Transcription-3 (p-STAT3) in ER+ breast cancer cells exposed to FSS. **a**) Quantification and distribution of normalized fluorescence in single cells coupled with one-way ANOVA showing statistically significant changes between the sheared population and non-sheared population stained for p-mTOR. **b**) Western blot assay for p-mTOR (Ser2448). **c**) Graphical representation of the normalized band density. **d**) Quantification and distribution of normalized fluorescence in single cells coupled with one-way ANOVA showing statistically significant changes between the sheared population and non-sheared population stained for p-STAT3. **e**) Western blot assay for p-STAT3 (Tyr705). **f**) Graphical representation of the normalized band density. The amount of phosphoprotein was normalized to Rho GDIα for both p-mTOR and p-STAT3 and the amount of phosphoprotein from the non-sheared population was standardized to 1. Off–chip data is representative of n=2 biological replicates. (*** indicates statistically significant p<0.001, * indicates statistically significant p<0.05, ns indicates statistically non-significant p>0.05).

AKT phosphorylation is downstream of PI3K and is a crucial signal transduction network in the promotion of tumor initiation and progression.[30] mTOR is a downstream effector and regulator of AKT and targeting of the AKT/mTOR axis in metastatic breast cancer has been FDA approved.[35] The TAMRAD clinical study which focused on an mTOR inhibitor (Everolimus) in combination with Tamoxifen effects on HER2-, Aromatase Inhibitor-resistant metastatic breast cancer.[35] This study found that the combination of Tamoxifen and Everolimus increased both the clinical benefit rate and the time to progression over Tamoxifen alone.[36] The risk of death was also reduced by 55% in the combination group.[37] To gain greater insight on the AKT/mTOR signaling axis following exposure to FSS both, on-chip and off-chip experiments were performed to determine if there was an activation cascade of mTOR following AKT phosphorylation. Cells exposed to 10 dyn/cm^2^ FSS were found to exhibit enhanced phosphorylation of mTOR (Ser2448) in both single cell and bulk analysis (fold change 4.5±0.5 with p-value < 0.05) when compared to non-sheared cells (Figure 4A–C, Figure S5). Figure 4A shows a third quartile in the sheared population that is even more activated than the fourth quartile of the non-sheared population and more than 50 outliers hyperactivated. This is of special significance since mTOR is responsible for proliferation and survival in cancer cells, and as stated above, inhibitors of mTOR have shown clinical benefit in HR+/HER2- metastatic breast cancers that showed resistance to hormonal therapies alone.[37, 38] Furthermore, previous studies have described the relationship between phosphorylation of PI3K/AKT/mTOR pathway and address it as a promising therapeutic target.[39–41]

STAT3 via phosphorylation is downstream of epidermal growth factor receptors (EGFR) and cellular SRC Kinase (c-SRC) pathways, is overexpressed in ER^+^ breast tumors, and is associated with a more aggressive phenotype in terms of cell proliferation, survival, differentiation and apoptosis.[42]. Due to this, we next sought to evaluate changes in STAT3 phosphorylation following exposure to FSS. On-chip analysis showed a clear difference between the distribution of the fluorescence single cell response of MCF-7 cells stained for p-STAT3 exposed to 10 dyn/cm^2^ FSS compared to and non-sheared cells (Figure 4D, Figure S6). This suggests that a more aggressive phenotype observed in the secondary tumor could result from FSS-mediated overexpression of p-STAT3. In fact, almost the whole four quartiles of the non-sheared population have fluorescent signal intensities that are under the second quartile of the sheared population which suggests that the exposure to 10 dyn/cm^2^ is easily enhancing STAT3 phosphorylation. A similar result was observed in the off-chip analysis by western blot showing a 2.1 ± 0.7-fold increase in STAT3 phosphorylation (Figure 4E–F). STAT3 mediates qualities that would enhance metastatic colonization such as cell proliferation, survival, and angiogenesis.[43, 44] Understanding mechanisms that enhance p-STAT3 activity at secondary sites, such as exposure to FSS, is pivotal to hindering the metastatic spread of breast cancer. Furthermore, it is important to highlight that after phosphorylation of STAT3, it mediates changes in gene expression that are associated with increased tumor cell growth, invasion, and metastasis.[43, 45] Because of these findings, it is of interest to carry on more research on STAT3 as a promising therapeutic target since this protein has been linked to recurrence and aggressiveness at secondary sites and is now shown to be promoted by FSS.[42]

### Exposure to FSS does not alter estrogen receptor phosphorylation in sheared and non-sheared populations of cells.

**Figure 5.**
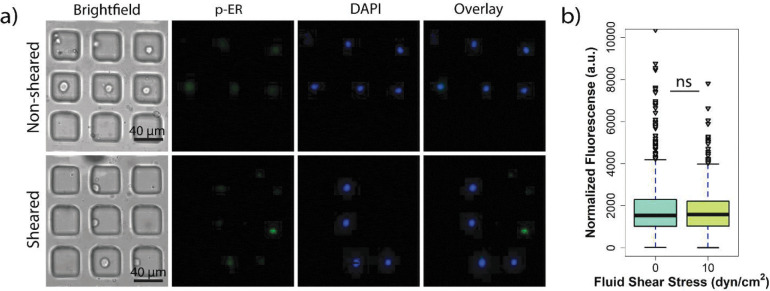
Exposure to FSS does not result in differences in p-ER levels between sheared and non-sheared MCF-7 cancer cells. **a**) Immunostaining images of p-ER expression in non-sheared cells and cells sheared at 10 dyn/cm^2^ **b**) Quantification and distribution of normalized fluorescence in single cells coupled with one-way ANOVA showing statistically nonsignificant changes between the sheared and non-sheared population (*** indicates statistically significant p<0.001, ns indicates statistically non-significant p>0.05).

Induction of cell proliferation and survival through the phosphorylation of AKT, mTOR, and STAT3 can result in downstream phosphorylation of estrogen receptor α (ERα) resulting in enhanced DNA binding and transcriptional activity.[46, 47] Recent studies have shown that hyperactivation of the PI3K/AKT/mTOR pathways drives tumorigenesis in ER+ breast cancer [48, 49] and can result in tumors becoming more aggressive.[50, 51] Based on these findings, it was important to investigate if the observed findings that exposure to FSS resulting in enhanced AKT, mTOR, and STAT3 phosphorylation also resulted in downstream of phosphorylation of ERα (Ser167). On-chip single cell immunostaining studies were performed to investigate if exposure to FSS altered the ERα phosphorylation between sheared and non-sheared populations of MCF-7 cells (Figure 5A–B). Similar to the ERK1/2 findings, there was no significant difference in ERα (Ser167) phosphorylation between the distribution of both populations (Figure 5C). In fact, both distributions depicted in Figure 5C show that the quartiles for these populations have the same fluorescence intensity levels. To confirm these results, it was found that 76% of the sheared and 73% of the non-sheared populations were positive for ER-α (Ser167), which suggests that effectively exposure to FSS does not enhance ER phosphorylation since there is no difference in the number of cells stained (Figure S7). This suggests that even though phosphorylation of AKT, mTOR and STAT3 is occurring, phosphorylation of ER at Ser167 is not taking place in the system, at least not immediately after shearing. Bulk analysis for p-ER by off-chip experimentation was also performed; however, western blot analysis failed to detect ERα phosphorylation. ER is part of a family of nuclear hormone receptors, and they play a role as ligand-activated transcription factors.[52] Because of this, future studies can look at other p-ER sites and so, this study may work as a framework. Since proteins upstream of p-ER Ser167 were being affected when exposed to FSS, it was expected that they may affect p-ER activity as well, but according to the results, in the time frame and phosphorylation site studied here, they are not. Two potential explanations for the lack of observable ERα phosphorylation at Ser167 could be the duration of the shearing or when the cells were interrogated for protein phosphorylation. It could be that the cells require a longer shearing duration or a grow-out period post-shear to induce ERα phosphorylation.

## Conclusions

This study demonstrated how exposure to FSS results in phosphor-protein changes in ER^+^ breast cancer cells, specifically enhanced phosphorylation of proteins in the AKT/mTOR signaling pathway (Figure S8). A unique aspect of these findings was the ability to perform both single cell analysis (on-chip experiments) and bulk analysis (off-chip experiments) using a modular microfluidic platform. The modular nature of the device resulted in cells being exposed to nearly uniform magnitudes of FSS to ensure that any observed heterogeneity in signaling was due to the cell itself and not the magnitude of shear it was exposed studies. On-chip and off-chip studies showed that Ki67, a protein produced during cell division and used as a marker for proliferation, was overexpressed in the sheared population compared to the non-sheared population suggesting that cells become more proliferative after being exposed to FSS. Furthermore, exposure to shear resulted in increased levels of protein phosphorylation of AKT, mTOR, and STAT3, all three of which are associated with enhanced cell proliferation and survival. Interestingly, there were no observed differences in the phosphorylation of ERK1/2 or ERα between the sheared and non-sheared populations of cells suggesting that exposure to shear induces select pathways. The single cell findings support the hypothesis that select cells exhibit extremely high levels of protein phosphorylation and Ki67 expression which could translate to CTCs that ultimately survive metastasis and go on to form more aggressive secondary tumors.

## Materials and Methods

### Design and fabrication of the shearing device

**Figure 6.**
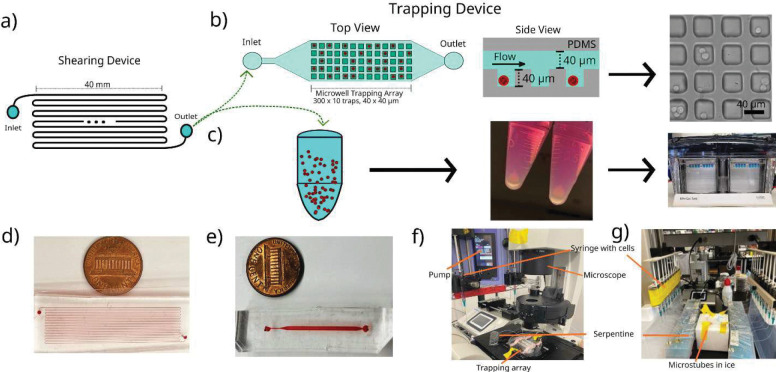
Workflow of the modular microfluidic device and experimental setup for on-chip and off-chip analysis of ER+ breast cancer cells exposed to FSS. **a**) Top view of the shearing device consisting of a 1 m long channel that is 70 μm wide and 100 μm tall. **b**) Top and side view of the microwell trapping array consisting of a 3000-member array of 40 × 40 μm wide and 40 μm deep traps used for single cell analysis as well as a representative brightfield image of MCF-7 cells after being sheared and trapped. **c**) Depiction of a microtube used to collect cells for off-chip analysis which are then pelleted and analyzed by western blot. **d**) Picture of the shearing module with red dye in the fluidic channel. **e**) Picture of the microwell trapping array with red dye in the channel. **f**) Real setup of an on-chip analysis experiment mounted on the microscope. **g**) Real setup of an off-chip analysis experiment showing multiple shearing modules depositing cells into microtubes.

The design and geometry of the microfluidic shearing device were made using Clewin Layout Editor (5.4.30.0 version, WieWeb software). Two shearing devices were created, one device consists of a single fluidic channel 1 m long, 70 μm wide, and 100 μm tall and it was used to shear at rates of 10 dyn/cm^2^. The other device was 1.5 m long, 100 μm wide and 150 μm tall and it was used to shear at rates of 20 dyn/cm^2^ (Figure 6A). The device geometry was fabricated on a silicon wafer using standard soft lithography methods. SU-8 2025 (a negative photoresist, Kakaku Advanced Materials) was deposited on a clean 4” wafer and baked for 30 min at 65°C and then at 95°C for 45 min. After cooling, the wafer was exposed to 260 mJ/cm^2^ of power intensity using a Microwriter ML3 Pro with no correction on focus to crosslink the SU-8. A post-exposure bake was performed using the same conditions as the pre-exposure bake. SU-8 developer solution (Kakaku Advanced Materials) was used to remove the uncrosslinked SU-8 and obtain the desired patterns followed by a final hard bake at 150°C for 30 min to increase wafer durability. The polydimethylsiloxane (PDMS, Sylgard 184, Ellsworth Adhesives) replicas used for the device were made by mixing a 10:1 ratio of base to curing agent followed by degassing in a vacuum chamber. The mixture was poured into the silicon master and was cured for 12 h at 65°C. Individual PDMS replicas were cut to size with an X-Acto knife, removed from the silicon master followed by the inlet and the outlet ports made using a blunted 18-gauge needle. Finally, the PDMS was bonded to a 25mm × 75mm glass slide (Corning) using an O2 Harrick Plasma PDC-32G basic plasma cleaner (Harrick Plasma, Ithaca, NY, USA) for 2 min and 30 s and then exposed to plasma for 15 s (Figure 6D).

### Design and fabrication of the microwell trapping array.

The geometry of the microwell trapping array was designed using Clewin Layout Editor (5.4.30.0 version, WieWeb software). The microwell trapping array is a two PDMS layer device with the bottom layer containing the microwell traps and the top layer containing the flow channel (Figure 6B). The trapping layer consists of an array of 3000 40 × 40 μm wide and 40 μm deep traps across 10 rows and 300 columns. The flow channel is 40 μm tall, 800 μm wide, and 3 cm long. The two silicon masters were fabricated using SU-8 2025 which was deposited on a clean 3” wafer and baked for 30 min at 65°C and then at 95°C for 30 min. After cooling, the wafers were exposed to 250 mJ/cm^2^ of power intensity using a Microwriter with no correction on focus to crosslink the SU-8 followed by a post-exposure bake as described above. SU-8 developer solution (Kakaku Advanced Materials) was used to remove the uncrosslinked SU-8 followed by a hard bake at 150°C for 30 min The top (fluidic) and bottom (trapping array) PDMS layers were plasma bonded using an O2 Harrick Plasma PDC-32G basic plasma cleaner (Harrick Plasma, Ithaca, NY, USA) for 2 min and 30 s and then exposed to plasma for 60 s. After exiting the plasma cleaner, the two layers were aligned and pressed together.

### Cell culture and reagents

MCF-7 human breast cancer cells (ER^+^/PR^+^) were acquired from American Type Culture Collection (Manassas, Va.). Cells were maintained with Dulbecco’s Modified Eagle Medium (DMEM) supplemented with 10% v/v HyClone Cosmic Calf Serum (Cytiva SH30087.03), 1% MEM Essential Amino Acids (Quality Biological Inc.), 1% MEM Non-Essential Amino Acids (Quality Biological Inc.), and 0.048 μg/ml Insulin (Insulin, Human Recombinant dry powder, Sigma Aldrich). Cells were maintained in either T-75 or T-182.5 (VWR #10062) flasks in a humidified incubator at 37°C and 5% v/v CO2. The cells were subcultured when 80–90% confluent by first washing the cells with 1X phosphate-buffered saline (PBS: 137 mM NaCl, 10 mM Na2HPO4, 27 mM KCl, and 1.75 mM KH2PO4 at pH 7.4) and then detaching the cells with 3 mL of 3.7 mM UltraPure^™^ EDTA solution, pH 8.0 (Thermofisher #15575020) diluted in 1X PBS before re-seeding into a new cell culture flask. Cells used for both on-chip and off-chip experimentation were at 80–90% confluence at time of experiment. Prior to exposure to FSS, cells were first washed with 1X PBS, and then detached with 2 mL of Accutase (Invitrogen) to prevent clumping of cells in the syringes and devices.

### On-chip experiments, immunofluorescent staining, and image analysis

On-chip experiments are defined as the shearing event and cell capture for single cell analysis using both the shearing device and microwell trapping array. Both devices were prepared by manually flowing extracellular buffer (ECB: 1.2 g HEPES, 8 g NaCl, 200 mg KCl, 420 mg MgCl2·6H2O, 265 mg CaCl2·2H2O, 1 g glucose dissolved in 1 L deionized water, pH 7.4) to remove the air followed by connecting the shearing device to the trapping array using ~5–7 cm of Tygon tubing (0.022” inner diameter × 0.042” outside diameter, Cole-Parmer). 15 cm of tubing was used to connect the inlet of the shearing device to a 23-gauge needle (BD precision, #305145) attached to a 1 mL syringe (BD Sciences) placed in a syringe pump 11 Pico Plus Elite (Harvard Apparatus, Cambridge, MA, USA) to apply the FSS on the MCF-7 cells (Figure 6A). 14 cm of tubing was used to connect the outlet of the microwell trapping array to a waste container to collect all expelled media and cells (as is shown in Figure 6B). To prevent clumping of the MCF-7 cells and facilitate single cell shearing, the cells were strongly pipetted before putting them into the syringe at a density of 1×10^6^ cells/mL. A constant flowrate of 7 μL/min was used to shear at 10 dyn/cm^2^ which represents an average residence time of 60 s using the 70×100 μm shearing device. When shearing at 20 dyn/cm^2^ the flowrate was 48 μL/min. For on-chip analysis, the cells exiting the shearing device were allowed to fill the microwell trapping array resulting in ~300–1000 trapped cells. Once isolated in the trapping array, the cells were allowed 45 min to settle in the individual traps followed by a 2 min wash with ECB at a rate of 10 μL/min prior to fixation. For every on-chip experiment performed, a companion non-sheared experiment was carried out by directly flowing the cells into the microwell trapping array followed by a similar 45 min on-chip incubation period to allow the cells to settle and ECB wash step. An image of the complete experimental set-up is shown in Figure 6F.

To perform on-chip immunostaining, the trapped cells were fixed by flowing a 4% paraformaldehyde (PFA) solution in PBS using a syringe pump at a rate of 8 μL/min for 2 h followed by a 20 min wash with PBS at a rate of 8 μL/min. After that, the cells were permeabilized by flowing Triton X 100 solution at 1% (w/v) in PBS at a rate of 4 μL/min for 12 h followed by a 20 min wash with PBS as described above. Blocking was performed by flowing a blocking buffer (0.5% w/v BSA in PBS) at a rate of 8 μL /min for 1 h. The immunostaining solution was made under sterile conditions by mixing 492 μL of BSA 0.25% (w/v) in PBS, 3 μL nuclear stain Hoechst 33342 (60 μM), and the corresponding antibody for the protein of interest. The antibodies (and their dilutions) were: p-ERα (Ser167) (5 μL, ratio 1:100, Cell Signaling Technologies, #64508), p-mTOR (Ser2448) (5 μL, ratio 1:100, Cell Signaling Technologies, #5536), p-STAT3 (Tyr705) (5 μL, ratio 1:100, Cell Signaling Technologies, #9145), p-ERK1/2 (Thr202/Tyr204) (5 μL, ratio 1:100, Cell Signaling Technologies, #9101), p-AKT (Ser473) (5 μL, ratio 1:100, Cell Signaling Technologies, #4060), and Ki67 (5 μL, ratio 1:100, Biolegend, #56–5698-82). Each stain solution was transferred into a 1 mL syringe and flowed through the device at a rate of 1 μL/min for 7 h at room temperature in the dark. The Ki67, p-AKT, p-STAT, and p-ERK antibodies were fluorescently labeled with GFP eliminating the need for a secondary antibody. For experiments using these antibodies, the device was washed with PBS as described above to remove any remaining stain. For the experiment with the p-ERα and p-mTOR, the primary antibody is not conjugated. 5 μL of the unconjugated primary antibodies was mixed with 495 μL of BSA 0.25% (w/v). The anti-antibody, Alexa Fluor 488 IgG (Invitrogen, #A11008), was flowed after flowing and washing (with PBS for 30 min) the primary antibody. The secondary antibody solution was prepared by mixing 492 μL of BSA 0.25% (w/v) in PBS, 3 μL nuclear stain Hoechst 33342 (60 μM), and 5 μL of Alexa Fluor 488. To image the cells, the trapping array was mounted on the stage of a fluorescent DMi8 inverted microscope (Leica Microsystems, Wetzlar, Germany) outfitted with a FITC filter (excitation λex: 460 to 500 nm, emission λem: 512 to 542 nm), DAPI filter (excitation λex: 325 to 375 nm, emission λem: 435 to 485 nm), and brightfield. All images were taken at 20X magnification. Once the images of the array were taken, ImageJ was used to analyze the fluorescent signal of every cell. To do so, the area of each cell was manually traced in ImageJ and then the fluorescent signal was quantified. After this, the same area was measured in the surrounding area of the cell to get the background signal and thus subtracted to get just the signal of the cell. Every experiment had at least 300 single analyzable cells.

### Off-chip experiments and western blot analysis

Off-chip experiments were performed using only the shearing device. A 10-syringe pump (KDS 220CE, KD Scientific) was used, allowing for an increased number of shearing modules. The device was set-up as described above, except that the device outlets were connected to a sterile 1.5 mL microcentrifuge tube via a 15 cm segment of Tygon tubing. Five shearing devices were prepared, and each device was connected to a 1 mL syringe. Since the shearing process takes about 3 hours, to prevent clumping of the MCF-7 cells and facilitate single cell shearing, the cells were diluted to 500,000 cells/mL (each syringe had 1 mL), and Pluronic ^™^ F-68 Non-ionic Surfactant (100X) (Thermofisher, #24040032) was added at 0.5% v/v. The cell suspension was flowed through the 70×100 μm shearing devices at a rate of 7 μL/min as described and collected at room temperature. Tubes were then collected every 45 minutes until all cells were exposed to shear which translates to 4 microtubes at the end of the shearing process. Every time one microtube was collected it was centrifuged to form a cell pellet, the media was removed, and cell pellets were frozen at −20 °C prior to performing the analysis. For western blot, cells were lysed using 150 μL of a mixture containing Mammalian Protein Extraction Reagent (M-PER) (Thermofisher 78501) with 1% protease (Thermofisher 1862209) and 1% phosphatase inhibitors (Thermofisher 1862495). The setup of off-chip experiments is shown in Figure 6G. The lysed cell pellets were centrifuged at 10,000 rpm at 4°C for 10 minutes. Then, a standardized amount of total protein, no less than 20μg, was added to each well. Reducing agent (Life Technologies B0009) and NuPAGE LDS sample buffer (Life Technologies B0007) were added to the samples per manufacturer’s protocol. Then, the proteins were heat denatured at 100°C for 10 minutes. The Bis-Tris-nuPAGE gel (Invitrogen, Grand Isles NY) was run in the Invitrogen mini gel tank (A25977) at 100V for 1 hour. Protein was transferred to nitrocellulose using iBlot and iBlot transfer stacks per manufacturer’s protocol (Invitrogen, Grand Isles, NY). The blots were blocked by incubation in EveryBlot blocking buffer (BIO-RAD 12010020) per manufacturer’s protocol. After blocking, the membrane was incubated with primary antibody overnight at 4°C for p-AKT (Ser473) (Cell Signaling Technologies, #4060), p-ERα (Ser167) (Cell Signaling Technologies, #64508), p-mTOR (Ser2448) (Cell Signaling Technologies, #5536), p-STAT3 (Tyr705) (Cell Signaling Technologies, #9145) and p-ERK1/2 (Thr202/Tyr204) (Cell Signaling Technologies, #9101). All primary antibodies were diluted 1:1000 in EveryBlot. The membrane was washed in 1X TBS-T three times for ten minutes each and incubated for 1 hour in Goat anti-rabbit IRDye^®^ 800CW secondary antibody (LI-COR Bioscience, Lincoln NE, #925–32211) at room temperature (1:10,000 dilution in EveryBlot) followed by three ten-minute washes in 1X TBS-T. Band density was determined by a LI-COR Odyssey imager. Loading control was to mouse monoclonal Rho GDI-α (Santa Cruz Biotechnology, Santa Cruz, CA sc-373724) diluted 1:500 in EveryBlot. Donkey anti-mouse IRDye^®^ 800CW secondary antibody (LI-COR Bioscience, Lincoln NE, #925–32212). Normalization was to loading control, N=2 biological replicates.

### Statistical Analysis

On-chip data presented in this study were representative of at least three independent experiments. The statistical difference between different groups was determined by the one-way ANOVA and statistical difference between groups was determined through the Tukey test using R Studio. A p-value < 0.05 was considered statistically significant (*) and p < 0.01 was considered statistically very significant (***), while p > 0.05 was considered statistically non-significant (ns). Off-chip data presented here consists of two biological replicates. The statistical significance, with a t-test, and graphical representations of band intensity between sheared and non-sheared populations were generated using GraphPad Prism. A p-value < 0.05 was considered statistically significant (*) and p > 0.05 was considered statistically non-significant (ns).
